# Functionality of Physical Activity Referral Schemes (PARS): A Systematic Review

**DOI:** 10.3389/fpubh.2020.00257

**Published:** 2020-06-25

**Authors:** Francis Ali Albert, Melissa J. Crowe, Aduli E. O. Malau-Aduli, Bunmi S. Malau-Aduli

**Affiliations:** ^1^College of Medicine and Dentistry, James Cook University, Douglas, QLD, Australia; ^2^Division of Tropical Health and Medicine, James Cook University, Douglas, QLD, Australia; ^3^College of Public Health, Medical and Veterinary Sciences, James Cook University, Douglas, QLD, Australia

**Keywords:** physical activity, exercise, referral schemes, primary healthcare practitioner, exercise specialists, patient health outcomes

## Abstract

**Background:** Physical activity (PA) is vital to maintaining good health. However, WHO estimates that 60% of the world's population are inadequately active. To enhance PA, Physical Activity Referral Schemes (PARS) have been established by some countries.

**Objective:** This study examined the functionality of the PARS process across different countries.

**Methods:** This systematic review was performed and reported in accordance with the PRISMA guidelines. Sixteen electronic databases were searched from January 1990 to May 2020. PARS studies, published in English language and in peer-reviewed journals, that reported adherence, outcomes, disease conditions, interventions, facilitators and barriers, were included in this review.

**Results:** Twenty-seven studies conducted across eight countries met the inclusion criteria. Most patients were referred for sedentary/inactivity reasons and supervised group-based activities was the most used intervention. Participants' average adherence rate was 77.5%. Adherence was either facilitated or hindered by type of support provided during and after intervention period. Inclusion of PA allied health specialist in the intervention enhanced positive health outcomes.

**Conclusion:** PARS is a key driver and motivator for individuals to undertake and adhere to PA interventions. Utilization of guidelines on evidence-based interventional PA for different types of diseases, effective use of common group supervised activities and the involvement of PA specialists may aid PA adherence and foster positive health outcomes.

## Introduction

Physical activity (PA) is vital to maintaining good health (1, 2). Furthermore, PA contributes to the prevention, management and treatment of non-communicable diseases including cardiovascular diseases, diabetes, stroke, colon and breast cancers, osteoarthritis, osteoporosis, obesity, and mental and psychological illnesses (3–5). Despite these benefits, the World Health Organization (WHO) estimates that 60% of the world's population fail to meet the recommended levels of PA (150 min of moderate-intensity PA per week or 10,000 steps per day) to confer relevant health benefits. Physical inactivity has been identified as a major problem in breast and colon cancer (20–25%), diabetes (27%) and ischemic heart (30%) diseases worldwide (1).

Primary care settings provide accessibility to healthcare for the majority of the population (6) and have therefore been utilized by various countries in the development of interventions that promote PA (7). Advice from primary healthcare professionals has been reported to significantly increase levels of PA (15–45% increase in self-reported PA) among patients (8). To sustain this increase, more than 360 min of patient contact time is required (9). Furthermore, estimated figures from past studies show that primary care physicians would need an extra 444 min per day to implement effective PA interventions (10). Considering the existing work load and time constraints on primary care physicians, effective PA counseling seems impractical (11). Consequently, there are divergent views regarding the effectiveness of PA counseling provided by General Practitioners (GPs) (9, 12, 13).

Referral of physically inactive patients to allied health professionals, such as: exercise physiologists (EP), physiotherapists, nutritionists and other PA specialists for individualized PA programmes could help fill this gap (14, 15). The intervention usually commences with referral of an eligible patient (who is mostly sedentary, at risk of developing or has a non-communicable disease) by health professionals like GPs and nurses to allied health professionals or community PA advisors for individualized PA programmes which include PA counseling and advice with prescriptions of moderate to vigorous aerobic exercises (16, 17). PA referral programmes typically last 10–12 weeks and have been established in primary care settings in various countries. However, the name, structural and implementation processes vary, depending on the country where the programme is delivered (18–21).

Referral schemes were first introduced in the United Kingdom during the 1990s, and now have well-established guidelines published by the National Institute for Health and Care Excellence (NICE) (20–22). Subsequently, similar referral programmes were introduced in other European countries as well as in Canada, New Zealand, and USA; and are often known as exercise referral schemes (ERS), physical activity on prescription (PAP) or physical activity referral scheme (PARS) (23–25). In Australia, they are predominantly called chronic disease management (CDM) and were introduced into the Medicare system in 2006 (26). Nonetheless, for the purpose of this review paper, the referral schemes will be addressed as Physical Activity Referral Schemes (PARS).

Previous studies have expressed doubts over the effectiveness of PARS (5, 27) due to reported limited uptake of the interventional programmes and non-sustainability of PA gains (28–30). For instance, a low national CDM consultation rate of 0.26% (31) and only 1% of consultations by GPs were reported in Australia (12). Systematic reviews on the effectiveness of referral schemes have shown that the programmes fostered increased PA levels in overweight, non-sedentary and elderly individuals, but the gains were not sustained after 1 year (20, 32, 33). Williams et al. (5) assessed the effectiveness of primary care-initiated PARS in improving long-term participation of sedentary adults. The study concluded that PARS has a small effect in increasing PA in sedentary adults and suggested that future PARS should concentrate on how to improve uptake and adherence. Pavey et al. (20) assessed the impact of PARS on PA and health outcomes and concluded with doubts on the effectiveness of PARS for improving PA, fitness or health indicators. Most of the reviews to date have been limited in scope, majorly focusing on quantitative studies, particularly randomized control trials (RCTs) (29, 34–36), and only few reviews have evaluated qualitative studies (22). Of great consequence is the fact that majority of the reviews have been focused on single countries. Given that PARS have been established in many developed countries, it will be beneficial to obtain a multinational and generalizable perspective on their effectiveness. Thus, systematic evaluation of the functionality of PARS, within a wider context, is significant to understanding their benefits and participants' responses to the intervention, particularly in relation to the referral process, barriers to adherence; support mechanisms utilized to foster adherence and health outcomes.

This multinational review therefore aimed to examine the functionality of PARS by investigating the influence of type of disease and intervention on uptake and health outcomes as well as patients' perceptions of motivators and barriers to effective PARS processes. This review addressed the following research questions:

- How does type of disease and intervention influence adherence and health outcomes?
- What are participants' views on the facilitators and barriers to attaining intervention goals?

## Methods

The systematic review was conducted and reported in accordance with the PRISMA (Preferred Reporting Items for Systematic Reviews and Meta-Analyses) Statement (37).

### Inclusion and Exclusion Criteria

There was no restriction on study design. Studies where participants were advised/counseled on PA in a single contact or referred by a health professional (e.g., a GP or nurse) to an allied health professional (e.g., physiotherapist or EP) were included. Studies were included if they were written in English, published in peer-reviewed journals between 1990 and 2020—considering that referral schemes were first introduced in the 1990s, included adult study participants who were older than 18 years, examined the PARS process. Also, the study must have reported the following outcome measures: Disease conditions (reason for referral/disease characteristics of referred of participants), type of intervention, health/PA related outcome of intervention, adherence rate, and facilitators and barriers to effective intervention programmes.

Studies were excluded if they did not report the above characteristics or were literature reviews, used to check the psychometric characteristics of instruments, opinion papers, national guidelines, reports, used to examine the PARS process from the perspective of the physician and if data from other studies were used to model cost-effectiveness.

### Search Strategy

Electronic databases comprising Medline Ovid, Medline (Pubmed), Cinahl, Informit, Scopus, SportDiscus, Academic Search Complete, SpringerLink, ArticleFirst, Taylor & Francis, Wiley Online, SAGE, ScienceDirect, ProQuest, Embase, and The Cochrane Library were searched from January 1990 to May 2020. Text words and indexed terms included “exercise, physical activity, sport, walk, run, physical fitness, exertion, general practitioner, family physician, refer, secondary care, and exercise physiology.” The search strategy used is presented in Appendix 1. Reference lists from previous systematic reviews and included studies were also screened for relevant additional inclusions.

### Study Selection Process

**Figure 1:**
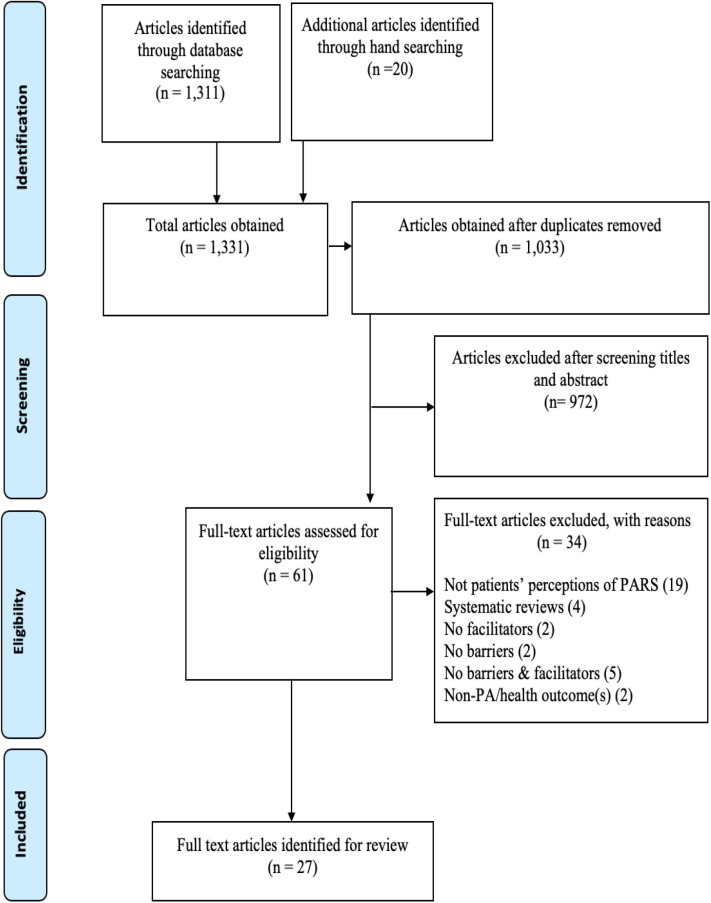
PRISMA flow chart of selection process.

The articles identified from all the databases were imported into Endnote X9.3 software, then titles and abstracts were screened. Two authors (FAA and BSMA) independently screened the titles and abstracts of the retrieved articles and excluded irrelevant ones. Subsequently, full-text articles categorized as potentially eligible for inclusion were screened in a consensus meeting and disagreements were resolved in real time until consensus was reached. Figure 1 portrays a detailed PRISMA flow diagram.

### Data Extraction and Analysis

Due to the heterogeneous nature of the included articles, meta-analysis was not possible (38). Study characteristics included study aims and country where study was conducted, study design, venue of the activity, who led the intervention and study participants. To further explore the functionality of the PARS program, the following characteristics were extracted: Disease conditions (reason for referral/disease characteristics), referrer, intervention and follow-up period, adherence rate/uptake, health outcomes, facilitators and barriers.

### Content Analysis

Inductive content analysis was employed for the eight qualitative studies included in this review to determine reoccurring themes (39). This analysis was carried out in three stages of coding, creating categories and abstraction. In Stage 1, one author (FAA) extracted data, listed all descriptions and developed coding frames for the following: disease conditions, intervention used and follow-up, health outcome, adherence, facilitators and barriers to the PARS process. In Stage 2, two authors (FAA and BSMA) developed and discussed preliminary categories. In Stage 3, final categories were created and labeled by both authors. All discrepancies were evaluated and resolved in a consensus meeting. Validation and potential extension of the coding frame was made possible by replication test (reoccurring themes).

### Risk of Bias Assessment

The methodological quality of the included studies were assessed using the Quality Assessment Tool for Studies with Diverse Designs (QATSDD) (40). This tool contains 16 items and is used for examining studies with different research designs. The QATSDD tool was modified by excluding two criteria, “evidence of user involvement in design” and “statistical assessment of reliability and validity of measurement tool(s),” as they were not relevant to the included studies. The grading system used assessed each reviewed study on a scale of 0–3 for each criterion, with 0 = not at all, 1 = very slightly, 2 = moderately, and 3 = complete. The criteria scores were summed and expressed as a percentage of the maximum possible score to assess the methodological quality of included studies. For ease of interpretation, the percentage scores were classified into low (<50%), medium (50–80%), or high (>80%) quality evidence. The criteria included were (1) theoretical framework; (2) aims/objectives; description of research setting; (4) sample size; (5) representative sample of target group, (6) procedure for data collection; (7) rationale for choice of data collection tool(s); (8) detailed recruitment data; (9) fit between research question and method of data collection (Quantitative only) (10) fit between research question and data collection method (Qualitative only) (11) fit between research question and method of analysis; (12) good justification for analytical method selected; (13) reliability of analytical process (Qualitative only); (14) strengths and limitations. An interpretation of the scores then allowed for classification into low (<50%), medium (50–80%), or high (>80%) quality evidence.

## Results

### Study Selection

After screening 1, 331 titles and abstracts and reviewing 61 full texts; 27 studies were selected for inclusion in the review (Figure 1).

### Characteristics of the Included Studies

Appendix 2 presents the summary characteristics of participants in the included studies. PA interventions were administered at primary healthcare centers in about half (48%) of the studies. Participant numbers ranged from 9 to 4, 317 and their mean ages ranged from 44.5 ± 15.7 to 82.0 ± 4.6 years. More females (65%) than males (35%) were referred for PA interventions. Table 1 presents information on reported disease conditions, interventions, adherence rates, health outcomes, facilitators and barriers for each reviewed study. The included studies originated from eight countries: UK (*n* = 13), Spain (*n* = 4), Sweden (*n* = 3), Denmark (*n* = 2), Australia (*n* = 2), Netherlands (*n* = 1), New Zealand (*n* = 1), and USA (*n* = 1). The study designs included RCTs (44.4%, *n* = 12), qualitative (29.6%, *n* = 8), longitudinal (7.4%, *n* = 2), case study (3.7%, *n* = 1), cohort study (3.7%, *n* = 1), mixed methods (3.7%, *n* = 1), cross sectional studies (3.7%, *n* = 1), and prospective (3.7%, *n* = 1).

**Table 1:** Key findings and frequency of occurrence.

**Country of study and study references**	**Study design and study references**	**Disease conditions (reason for referral/disease characteristics of referred patients) and study references**	**Intervention used and study references**	**Adherence/uptake**	**Facilitators, study references, and sample comment**	**Barriers, study references, and sample comment**
UK (48.1%, *n* = 13) (23, 30, 41–50)	RCTs (*n* = 12) (15, 17, 18, 28, 36, 41, 46, 47, 51–54)	Sedentary behavior/inadequately active (*n* = 16) (15, 17, 18, 23, 28, 30, 36, 41–44, 51, 52, 55–57)	Common group supervised activities (*n* = 12) (30, 36, 41–43, 45, 46, 48, 52, 53, 56, 58)	High adherence = (*n* = 17, 63%) (17, 18, 23, 28, 42–44, 46, 48, 50, 51, 53–55, 57–59)	Support during and after the scheme (*n* = 20) (15, 17, 18, 23, 28, 30, 32, 36, 42–46, 49–51, 53, 55, 56, 59) “If I could go with my husband, I would find the time”	Time constraints (*n* = 17) (15, 30, 32, 41–43, 45, 47–49, 51, 53–58) “I need different times, you know, that's what I do need”
Spain (14.8%, *n* = 4) (17, 51–53)	Qualitative (*n* = 8) (42–45, 48, 50, 58, 59)	Cardiovascular diseases (*n* = 13) (18, 23, 32, 41–43, 45–47, 53, 55, 56, 59)	PA counseling and advice (*n* = 10) (15, 17, 23, 28, 41, 46, 47, 50, 51, 55)	Medium adherence (*n* = 6, 22.2%) (15, 41, 45, 49, 52, 56)	Participant's goals and motivation (*n* = 14) (15, 17, 28, 36, 42, 44, 46, 48, 50, 51, 53, 55–57) “My main aim is to stay fit, and active, and mobile”	Psychological/perceived negative feelings (*n* = 13) (30, 41, 44–49, 52, 53, 55, 57, 59) “Anxious, scared, it was due to seeing young fit and healthy males showing off and felt intimidated”
Sweden (11.1%, *n* = 3) (23, 36, 55)	Longitudinal (*n* = 2) (23, 56)	Overweight/obesity (*n* = 11) (18, 23, 32, 41, 42, 44, 45, 47, 48, 53, 56)	Individualized and supervised activities (*n* = 9) (18, 23, 28, 44, 45, 50, 54, 55, 59)	Low adherence (*n* = 4, 14.8%) (30, 32, 36, 47)	Professional advice and supervision (*n* = 10) (17, 18, 28, 36, 51, 53–56, 58) “The thought that there would be somebody who could actually advise me on what to do, so I didn't knacker myself I wouldn't dare try it by myself”	Unwell (*n* = 11) (15, 17, 18, 23, 28, 41, 42, 45, 54–56) “… caught virus, affected my heart and lungs and went to hospital.”
Australia (7.4%, *n* = 2) (15, 54)	Case evaluation (*n* = 1) (32)	Musculoskeletal/Aging reasons (*n* = 8) (18, 23, 32, 41, 42, 45, 49, 53)	Referred to other health professionals (*n* = 8) (15, 28, 32, 42, 47, 49, 55, 56)			
Denmark (7.4%, *n* = 2) (46, 56)	Cohort (*n* = 1) (49)	Diabetes (*n* = 6) (18, 23, 45, 46, 53, 56)	Self-administered PA (*n* = 4) (15, 18, 47, 51)			Inaccessibility–Transport/venue location (*n* = 11) (18, 23, 30, 41–43, 45, 47, 50, 55, 58) “There's no direct bus”
USA (3.7%, *n* = 1) (28)	Mixed methods (*n* = 1) (30)	Psychological illness (*n* = 6) (18, 32, 41, 45, 55, 58)	Individualized PA prescription without supervision (*n* = 4) (23, 36, 49, 57)		Incentives (*n* = 8) (36, 41, 43, 45–47, 58, 59) e.g., giving 10 pounds gift vouchers	
Netherlands (3.7%, *n* = 1) (18)	Prospective (*n* = 1) (56)	At-risk smoker (*n* = 5) (28, 42, 47, 53, 56)			Social engagement with other participants (*n* = 5) (41, 48, 49, 52, 55) “And I found the whole process valuable, particularly going along with other people who had similar problems and sharing their problems with them”	Inadequate support (*n* = 10) (15, 30, 32, 41, 42, 45–47, 55, 59) “After quite a few weeks of not seeing him the counselor, that I slipped back a bit”
New Zealand (3.7%, *n* = 1) (57)	Cross sectional study (*n* = 1) (57)	Cancer (*n* = 1) (54)				
		Stroke (*n* = 1) (50)				Financial constraints (*n* = 4) (30, 36, 48, 55) “They charge money and its expensive”

*This table provides information on the findings from the key variables of the reviewed studies and their frequency of occurrence*.

*Country of study and study references: This column shows the eight countries where the studies originated from, the frequency of studies per country and their reference number*.

*Study design and study references: This column shows the study design employed in each of the studies, the frequency of each design used among the reviewed studies and their reference number*.

*Disease conditions (reason for referral/disease characteristics of referred patients) and study references: This column shows the reason for referral or the disease characteristic of participants as reported by each study, the frequency of occurrence for each disease group among the reviewed studies and their reference number*.

*Intervention used and study references. This column shows the intervention(s) reportedly used by each study, their frequency of occurrence among the reviewed studies and their reference number*.

*Adherence/uptake: This column shows the reported adherence of participants to study interventions goals, their frequency of occurrence among the reviewed studies and their reference number*.

*Facilitators, study references, and sample comment: This column shows the reported facilitators motivating adherence to interventions goals, their frequency of occurrence among the reviewed studies, their reference number and a sample comment reportedly made by a participant to support this facilitator*.

*Barriers, study references, and sample comment: This column shows the reported barriers preventing adherence to interventions goals, their frequency of occurrence among the reviewed studies, their reference number and a sample comment reportedly made by a participant to support this barrier*.

### Disease Conditions, Reason for Referral, and Disease Characteristics of Referred Participants

Table 1 provides information on frequency of occurrence of key findings. More studies were conducted in the UK (48.1%) compared to other countries and were mostly RCTs. Disease conditions (reason for referral/disease characteristics of participants) were clustered into nine categories. Sedentary/inactive reasons recorded the highest number of referral with sixteen (59.2%), of the twenty seven included studies referring participants to PA programmes for sedentary/inactive behavioral reasons (15, 17, 18, 23, 28, 30, 36, 41–44, 51, 52, 55–57). Referral for cardiovascular disease related reasons was recorded in thirteen (48.1%) studies (18, 23, 32, 41–43, 45–47, 53, 55, 56, 59), other reasons for referral were overweight/obesity (40.7%) (18, 23, 32, 41, 42, 44, 45, 47, 48, 53, 56), musculoskeletal/aging reasons (29.6%) (18, 23, 32, 41, 42, 45, 49, 53), diabetes related reasons (22.2%) (18, 23, 45, 46, 53, 56), psychological illness (22.2%) (18, 32, 41, 45, 55, 58), at-risk smokers (18.5%) (28, 42, 47, 53, 56), people with diagnosis of cancer (3.7%) (54), and stroke (3.7%) (50). The major (80%) referrers were GPs, however, few studies reported other health care professionals (dietitians, nurses and physiotherapists) as the referrer.

### Intervention, Adherence, and Health Outcomes

Criteria for measuring the success of the PARS process in this review included the intervention used, adherence/uptake by the participants and the reported health/PA related outcomes. As shown in Table 1, the interventions reportedly used in the management of chronic diseases across different countries included: common group supervised activities which was reported in twelve studies (44.4%) (30, 36, 41–43, 45, 46, 48, 52, 53, 56, 58), PA counseling and advice (37%) (15, 17, 23, 28, 41, 46, 47, 50, 51, 55), individualized and supervised activities (33.3%) (18, 23, 28, 44, 45, 50, 54, 55, 59), referral to other health professionals (29.6%) (15, 28, 32, 42, 47, 49, 55, 56), self-administered PA (14.8%) (15, 18, 47, 51), and individualized PA prescription without supervision (14.8%) (23, 36, 49, 57).

Table 2 shows the disease conditions patients were referred for, the interventions reportedly used in the management of these diseases and the outcome(s) recorded for each intervention. For sedentary/inactivity behavioral reasons (15, 17, 18, 23, 28, 30, 36, 41–44, 51, 52, 55–57), counseling/advice (15, 17, 23, 28, 41, 51), and common group supervised activities (30, 36, 41, 43, 52, 56) were the most reported interventions (*N* = 6 for each activity); while individualized PA prescription without supervision (36) was the least reported intervention (*N* = 1). All the interventions reportedly recorded positive outcomes for the participants. Among the studies which reported the referral of participants for cardiovascular disease related reasons (18, 23, 32, 41–43, 45–47, 51, 53, 56, 59), counseling/advice (23, 45–47, 51), and common group supervised activities (41, 42, 46, 53, 56) were the most reported (*N* = 5 each) interventions; while no study reported the use of individualized PA prescription without supervision. There were positive outcomes for all the interventions.

**Table 2:** Relationship between disease conditions, intervention used and outcome of intervention.

**Disease condition (reason for referral/characteristics of referred patients)**	**General intervention used and study reference number**						**Outcome(s) recorded after intervention**					
	**C/A**	**SAPA**	**IPAWS**	**ROHP**	**CGSA**	**IS**		**SAPA**	**IPAWS**	**ROHP**	**CGSA**	**IS**
Sedentary behavior (insufficiently active) *N* = 16 (15, 17, 18, 23, 28, 30, 36, 41–44, 51, 52, 55–57)	(15, 17, 23, 28, 41, 51)	(15, 18)	(36, 57)	(15, 42)	(30, 36, 41, 43, 52, 56)	(18, 23, 28, 44)	Positive outcome	(15, 17, 23, 28, 41, 51)	(36, 57)	(15, 28, 42)	(30, 36, 41, 43, 52, 56)	(18, 23, 28, 44)
							No effect	Nil				
Cardiovascular diseases *N* = 13 (18, 23, 32, 41–43, 45–47, 51, 53, 56, 59)	(23, 45–47, 51)	(18, 47)		(32, 47)	(41, 42, 46, 53, 56)	(18, 43, 47, 59)	Positive outcome	(18, 23, 41, 46, 47, 51)	(23, 47)	(41, 42, 46, 53, 56)	(41, 42, 46, 53, 56)	(18, 43, 47, 59)
							No effect	Nil				
Overweight/obesity *N* = 11 (18, 23, 41, 42, 44, 45, 47, 48, 53, 56)	(23, 41, 53)	(18, 47)		(32, 47)	(41, 42, 45, 48, 53, 56)	(18, 23, 44, 45)	Positive outcome	(18, 23, 41, 47, 53)		(47, 51)	(41, 42, 45, 48, 53, 56)	(18, 23, 44, 45)
							No effect	Nil				
Musculoskeletal/aging reasons *N* = 8 (18, 23, 32, 41, 42, 45, 49, 53)	(23, 53)	(18)	(23, 41)	(18, 32)	(42, 48)	(18, 45, 49)	Positive outcome	(18, 23, 53)	(23, 53)	(18)	(41, 42, 48)	(18, 45, 53)
							No effect			(32)		
Diabetes *N* = 6 (18, 23, 45, 46, 53, 56)	(23, 46)	(18)	(23)		(45, 46, 53, 56)	(46)	Positive outcome	(23, 46)	(23)		(45, 46, 53, 56)	(18, 45)
							No effect	Nil				
Psychological illness *N* = 6 (18, 32, 41, 45, 55, 58)	(32, 45)	(18)		(28, 32)	(41, 58)	(18, 55)	Positive outcome	(32, 45)		(28)	(41, 58)	(18, 55)
							No effect	18		(32)		
At-risk smoker *N* = 5 (28, 42, 47, 53, 56)	(28)	(47)		(28, 47, 56)	(42, 53)		Positive outcome	(47)		(28, 56)	(42, 53)	
							No effect	(28)		(47)		
Cancer *N* = 1 (54)				(54)			Positive outcome			(54)		
							No effect	Nil				
Stroke *N* = 1 (50)						(50)	Positive outcome					(50)
							No effect	Nil				

*This table provides information for the relationship between patient disease conditions, the interventions reportedly used in the management of these diseases and the outcome(s) recorded for each intervention*.

*Disease condition (reason for referral/characteristics of referred patients): This column shows the reason for referral or the disease characteristic of participants as reported by each study, the frequency of occurrence for each disease group among the reviewed studies and their reference number*.

*General intervention used and study reference number: This column is a collection of all the intervention reportedly used by the reviewed studies, the frequency of occurrence for each intervention group among the reviewed studies and their reference number. The intervention reportedly used in the reviewed studies included Counseling/advise (C/A); Self-administered PA (SAPA); Individualized PA prescription without supervision (IPAWS); Referred to other health professionals (ROHP); Common group supervised activities (CGSA); and Individualized and supervised activities (IS)*.

*Outcome(s) recorded after intervention: This column is a collection of all the outcomes reportedly recorded by each intervention in the reviewed studies, the frequency of reported outcome for each intervention group and their reference number. The first column under the outcome column shows if the intervention used reportedly had a positive effect or not while each row under that shows the reference for each study with this outcome. Participants who were reportedly counseled/advised went onto the other interventions. Therefore, the outcome for studies which reportedly used counseling/advice (C/A) as an intervention could be found in any of the other five outcomes for the following interventions SAPA, IPAWS, ROHP, CGSA, and IS*.

*C/A, Counseling/advise; SAPA, Self-administered PA; IPAWS, Individualized PA prescription without supervision; ROHP, Referred to other health professionals; CGSA, Common group supervised activities; IS, Individualized and supervised activities*.

For overweight/obese referrals (18, 23, 32, 41, 42, 44, 45, 47, 48, 53, 56), common group supervised activities (41, 42, 45, 48, 53, 56) was the most reported intervention (*N* = 6), counseling/advice (23, 41, 53), and individualized and supervised activities (18, 23, 45) were reported by three studies each, two studies each reported the use of self-administered PA (18, 47) and referral to other health professionals (32, 47). For musculoskeletal/aging reasons (18, 23, 32, 41, 42, 45, 48, 49), individualized and supervised activities (18, 45, 49) was the most reported intervention (*N* = 3); while other interventions were reported by two studies each except self-administered PA which was reported by only one study (18). All the interventions reported positive outcomes except one which reported the referral of participants to other health professionals (32).

Six studies each reported the referral of participants for diabetes (18, 23, 45, 46, 53, 56) and psychological illness (18, 32, 41, 45, 55, 58) related reasons. Common group supervised activities (45, 46, 53, 56) was the most reported (*N* = 4) intervention for the diabetic patients; while no study reported the use of referral to other health professionals. For psychological illness related referrals, one study (18) reported the use of self-administered PA, non for individualized PA prescription without supervision; while all other interventions were reported by two studies each. All the studies reported positive outcomes for the diabetes related referrals. For psychological illness, three of the five interventions used reported positive outcomes while self-administered PA and one (32) study which reported the referral of participants to other health professionals had no effect. However, when self-administered PA was combined with individualized and supervised activities in the same study, a positive outcome was reported (18). Referral to other health professionals (28, 47, 56) was the most reported (*N* = 3) intervention for at-risk smoking reasons (28, 42, 47, 53, 56). Common group supervised intervention (42, 53) was reported by two studies, one each for counseling/advice (28) and self-administered PA (47) and none for the remaining interventions. No effect was reported for participants who self-administered PA and also for one of the studies (47) which referred participants to other health professionals. However, two studies each reportedly had positive effects from common group supervised activities (42, 53) and those referred to other professionals (28, 56). Furthermore, when counseling/advice was combined with referral to other health professionals in the same study, a positive outcome was reported (28).

The study on cancer (54) reported the referral of participants to other health professionals while the study on stroke (50) reported the use of individualized and supervised activities as interventions. Both studies recorded positive outcomes for participants.

Adherence was defined as the proportion of participants who started and ended the PA referral programme. Studies with 75–100% adherence were categorized as having high adherence (17, 18, 23, 28, 42–44, 46, 48, 50, 51, 53–55, 57–59), 50–75% as having medium adherence (15, 41, 45, 49, 52, 56), and below 50%, were categorized as low adherence (30, 32, 36, 47). Table 1 depicts that overall, there was a positive adherence of 85.2% (high + mid adherence), while Table 2 shows that over 90% of the studies recorded positive health outcomes (examples include: enhanced PA, improved physical and mental health). Majority of the participants recorded notable health or PA outcome in the referral process except those referred for smoking reasons and some participants with musculoskeletal conditions who were referred to other professionals (28, 32, 47). In addition, two studies which examined the effects of ERS on cancer and stroke, designed individualized programmes for participants and were supervised by other healthcare professionals (EPs and physiotherapist, respectively). These studies reported positive health outcomes with high adherence by the participants (50, 54).

### Facilitators and Barriers

Facilitators and barriers to effective PARS process were categorized into five and six broad themes, respectively (Table 1). Five factors were identified as facilitators: perceived support: (15, 17, 18, 23, 28, 30, 32, 36, 42–46, 49–51, 53, 55, 56, 59), defined goals and motivation: (15, 17, 28, 36, 42, 44, 46, 48, 50, 51, 53, 55–57), professional advice and supervision during and after PARS programme: (17, 18, 28, 36, 51, 53–56, 58), incentives: (36, 41, 43, 45–47, 58, 59) and social engagement with other participants: (41, 48, 49, 52, 55). About half of the reviewed studies in which the participants reported perceived presence of support, development of personal goals and motivation, also recorded high or medium adherence and notable outcomes (17, 23, 28, 36, 42, 43, 45, 48–50, 53–56, 59). Some studies that provided professional counseling/advice as an intervention also had positive adherence and notable outcomes (17, 28, 36, 53, 55, 56, 58). Six (6) major factors were reported by participants as barriers. These included time constraints (15, 30, 32, 41–43, 45, 47–49, 51, 53–58), psychological/perceived negative feelings (30, 41, 44–49, 52, 53, 55, 57, 59), inaccessibility (transport/venue problems): (18, 23, 30, 41–43, 45, 47, 50, 55, 58), unwell (15, 17, 18, 23, 28, 41, 42, 45, 54–56), inadequate support (15, 30, 32, 41, 42, 45–47, 55, 59), and financial constraints (30, 36, 48, 55). Participants' views on the PA referral setting and accessibility were broadly categorized as scheme settings (leisure center or intervention environment) and accessibility (transport and distance to venue). Eleven out of the twenty-seven (40.7%) included studies considered this a barrier and two out of these eleven studies recorded low adherence rates (30, 47).

### Assessment of Methodological Quality

Based on the individual QATSDD assessment, results indicated that the scores ranged from 31 to 83% (Table 3). There were twenty medium quality studies (17, 18, 23, 30, 36, 42–45, 47, 48, 50–57, 59) compared to four high (15, 28, 41, 46) and three low quality studies (32, 49, 58). The low-quality studies had lower scores because they lacked a theoretical framework, had small sample sizes, poor reliability of analytical process, and poor description of strengths and limitations of the study. The studies with higher scores were RCTs and they were judged to be appropriate in their statistical analyses and trial designs.

**Table 3:** Quality assessment of the reviewed studies.

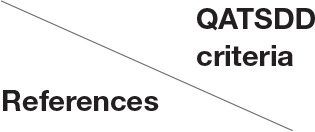	**1**	**2**	**3**	**4**	**5**	**6**	**7**	**8**	**9**	**10**	**11**	**12**	**13**	**14**	**Total score**	**% Maximum possible score**
James et al. (15)	2	3	3	1	2	3	3	3	2	n/a	2	3	n/a	3	30/36	83%
Grandes et al. (17)	0	2	3	3	3	3	3	3	0	n/a	0	3	n/a	1	23/36	64%
Gademan et al. (18)	0	3	1	0	1	3	3	3	2	n/a	0	3	n/a	3	22/36	61%
Lundqvist et al. (23)	0	3	3	1	1	3	2	3	0	n/a	3	3	n/a	3	25/36	69%
Ackermann et al. (28)	0	3	3	3	3	3	3	3	2	n/a	2	3	n/a	1	29/36	81%
Moore et al. (30)	0	3	1	3	3	2	3	3	0	2	3	2	1	3	29/42	69%
Dugdill et al. (32)	3	1	1	0	0	2	0	2	n/a	1	1	0	1	0	12/39	31%
Romé et al. (36)	1	3	1	2	2	3	2	3	2	n/a	2	2	n/a	3	25/36	72%
Grandes et al. (51)	0	2	3	2	3	3	3	3	0	n/a	0	3	n/a	3	25/36	69%
Isaacs et al. (41)	0	3	3	3	3	3	3	3	2	n/a	0	3	n/a	3	29/36	81%
Joyce et al. (42)	0	2	1	3	1	3	3	1	n/a	0	3	2	0	3	22/39	56%
Kallings et al. (55)	0	3	1	3	3	3	2	3	n/a	n/a	2	2	n/a	2	24/33	73%
Martin-Borras et al. (52)	0	3	3	2	3	3	2	2	2	n/a	0	3	n/a	3	26/36	72%
Roessler and Ibsen (56)	1	0	3	3	3	3	2	3	2	0	2	2	n/a	0	27/39	69%
Wormald et al. (43)	0	3	3	0	2	2	3	2	n/a	0	2	3	0	0	20/39	51%
Patel et al. (57)	1	3	2	1	2	3	2	2	2	n/a	2	2	n/a	3	25/36	69%
Eynon et al. (44)	2	3	3	2	2	2	1	2	n/a	3	2	3	3	1	29/39	74%
Gusi et al. (53)	0	2	3	3	2	3	3	3	0	n/a	0	3	n/a	3	25/36	69%
Hanson et al. (45)	1	3	3	1	1	3	3	3	n/a	0	3	3	1	3	28/39	72%
Shaw et al. (59)	1	3	3	2	2	3	3	3	n/a	0	2	2	0	2	26/39	67%
Sorenson et al. (46)	0	3	3	3	3	3	2	3	2	n/a	3	2	n/a	2	29/36	81%
Taylor et al. (47)	0	1	3	0	3	3	3	3	2	n/a	2	2	n/a	2	24/36	67%
Law et al. (48)	1	3	3	1	1	3	3	1	n/a	0	0	3	2	1	22/39	56%
Dinan et al. (49)	0	2	3	0	3	1	0	2	n/a	n/a	2	0	n/a	0	13/33	39%
Wormald and Ingle (58)	0	2	3	0	1	2	3	2	n/a	0	2	3	0	0	17/39	44%
Livingston et al. (54)	0	3	3	0	0	2	3	3	3	n/a	0	3	n/a	2	22/36	61%
Sharma et al. (50)	1	1	3	2	3	3	2	3	n/a	0	2	2	0	2	24/39	62%

*This table provides information on the quality assessment criteria used in this review*.

*QATSDD criteria: This row shows a list of all the Quality Assessment Tool for Studies with Diverse Designs (QATSDD) item employed in this review. The QATSDD item were numbered from one to fourteen. The interpretation of the numbers includes: (1) theoretical framework; (2) aims/objectives; (3) description of research setting; (4) sample size; (5) representative sample of target group, (6) procedure for data collection; (7) rationale for choice of data collection tool(s); (8) detailed recruitment data; (9) fit between research question and method of data collection (Quantitative only) (10) fit between research question and format and content of data collection method (Qualitative only) (11) fit between research question and method of analysis; (12) good justification for analytical method selected; (13) reliability of analytical process (Qualitative only); (14) strengths and limitations. The grading system used assessed each reviewed study on a scale of 0–3 for each criterion, with 0 = not at all; 1 = very slightly; 2 = moderately; 3 = complete; and n/a, not applicable as portrayed in the table*.

*References: This column shows all the reviewed studies and their year of publication listed according to their order in the references*.

## Discussion and Conclusion

### Discussion

This systematic review investigated the functionality of PARS process by exploring participants' disease conditions, interventions used, health outcomes and the facilitators and barriers to achieving intervention goals.

#### Referral Process, Study Designs, and Settings

Most of the studies in this review originated from the UK. This could be a result of the rapid expansion of PA programmes in the UK, its inclusion into the national policy and communities being allowed to operate various designs of the PARS programme (32). The low number of studies reported from the other countries indicate that there is paucity of research on the functionality of PARS in these countries and that further research is needed in this area. In terms of study design, quantitative research methods, particularly RCT dominated, while only one study employed the use of mixed methods design. This could be because the duration of PARS tend to be between 10 and 12 weeks (20, 60). Also, the “gold standard” in the evaluation of health pathway interventions is RCTs, however, they were limited by a short follow-up period. The average reported follow-up period in this review was 12 weeks (~3 months). Previous studies have highlighted the importance of considering studies with longer follow-up periods for the evaluation of the effectiveness of health pathway programs that aim to change participants' behavior (20). More studies employing the use of mixed methods are required to examine the functionality of PARS (30). This approach allows for triangulation between quantitative and qualitative research methods to uncover the best possible explanations for the observed phenomenon (61). Furthermore, mixed methods approach balances the flaws in either qualitative or quantitative research, is pragmatic and allows for triangulation of data which in turn fosters in-depth understanding and interpretation of convergent and divergent findings (62).

#### Disease Conditions, Interventions Used, and Outcomes

The current review found that sedentary/inactive participants were the most referred. This could be because, being “sedentary/inactive” is the frequent rationale offered by referrers for referring participants into PARS (63). Not all studies in this review that used PA counseling/advice as an intervention had positive outcomes, confirming the results of the systematic review by Pavey et al. (64), who showed that there was no difference in the proportion of individuals achieving improved PA outcomes after being advised by their GPs in comparison to other PA interventions. Most of the studies indicated short-term improvement in PA related outcomes like increased PA during leisure time but no effect was observed for other health related outcomes like overweight, cardiovascular disease and mental health (17, 51). This could be an indication that counseling and advice alone would not suffice to motivate participants to adhere to PA interventions and more supportive measures, such as professional supervision and engagement with other participants are required (45, 51). In a study where counseling and advice were combined with group-based supervised activities, there was an improvement in level of PA, cardiorespiratory, physical and mental health (41).

A comparison of reasons for referral in different countries showed that more patients with cardiovascular disease were referred into the PARS programme in the UK and that these participants were highly likely to participate and adhere to the PA referral programme when compared to other reasons for referral. This could be as a result of the prevalence of this disease and the popularity of the referral scheme as an alternative in the management of cardiovascular disease in the UK (32, 59). In addition, research has shown that PARS is effective in cardiac rehabilitation (65). However, some of the cardiovascular disease participants recorded low adherence rates and no outcome (32, 47). These participants were either referred to other professionals (32) or only counseled/advised (47) to participate in PA. A possible reason for the no outcome could be because adherence to the intervention dropped due to poor follow up (inefficient or lack of clinical reinforcements and support for participants) (51) implying that PA counseling and advice as the only intervention may not adequately motivate adherence to PA interventions.

Overweight/obese participants preferred common group activities and found it to be effective. These participants recorded positive outcomes and good adherence to study intervention goals (41, 42, 44, 48, 53, 56). Participants who were counseled/advised (18, 23, 41, 53, 54) recorded improved PA related outcomes after self-administered PA. However, there was low adherence for some of the participants (32, 47) hence, another disadvantage of this intervention. Participants who were referred to other health professionals recorded positive health outcomes but low adherence to interventions goals (32, 47). This could be because the overweight participants tend not to adhere to programme intervention goals because they believe that PARS is not appropriate for them (29).

All of the interventions used for participants referred for musculoskeletal/aging reasons (18, 23, 32, 41, 42, 45, 49, 53) resulted in positive outcomes and good adherence rates except for one study for which the outcome was not recorded (32). Participants who were advised/counseled to increase their PA adhered to the advice and recorded positive PA related outcomes (23, 41). Participants referred for diabetes related reasons (18, 23, 45, 46, 53, 56) recorded positive outcomes and good adherence rates. There was no difference in terms of outcome between the interventions used. Possible reason could be because participants' goal or disease conditions could act as a motivator toward achieving positive outcome for their disease conditions regardless of the intervention used (46, 53, 56). For participants with psychological illness (18, 32, 41, 45, 55, 58), most of the studies (41, 45, 55) reported positive outcomes except those studies in which participants reportedly self-administered PA (18) and one in which participants were referred to other health care professionals (32). Possible reason for poor health outcome could be the difficulty of adhering to intervention goals by participants with mental health conditions (29).

Some of the intervention used for participants with at risk smoking behaviors recorded positive outcomes and good adherence (28, 42, 47, 53, 56). The possible reason for this could be because more than one of the above interventions (referral to other health professionals and common group supervised activities) were combined and thus encouraged participants to improve the outcome of their disease (42, 53). Some other interventions used in these studies reportedly had no outcome (28, 47) and low adherence (47). Possible reason could be because of the type of intervention used coupled with the challenges of changing smoking habits (28, 47). Only one study each out of the 27 reviewed studies indicated the referral of cancer (54) and stroke patients (50) despite the positive effects of PA on stroke (66) and cancer (67). One possible reason could be the paucity of PA specialists (e.g., physiotherapists and EPs) in the management of such diseases which require highly skilled personnel. However, the study on cancer (prostate cancer) had positive outcome and mentioned the involvement of EPs in the management of the intervention (14). This further strengthened the reason for the positive outcome recorded by the participants. Hence, the need for more PA experts/specialists to manage PA interventions, especially for chronic and delicate diseases (15).

#### Facilitators and Barriers to Referral Process

Support from providers, peers, family and friends were identified as facilitators of participation, adherence and enhanced positive health outcomes for the participants; while the lack of these support networks was perceived as a barrier (47, 51, 68). Adequate supervision and follow-up support programmes by professionals reduced participants' anxiety and fostered motivation, while lack of on-going support was perceived as a barrier to uptake, adherence and sustained PA improvements (42). These findings have been previously reported by other studies (69, 70). Group activities and interactions with other participants also aided enjoyment of the PA referral programme (42, 50, 59). Involvement of EPs also facilitated better health outcomes for participants. Possible reason for this could be the professional advice and supervision provided by EPs (15). PARS that engage individuals in PA with practical, professional, supportive and follow-up measures are therefore required to obtain sustainable long-term gains (58).

Participants felt either intimidated or uncomfortable in unfamiliar environments (15, 29, 30, 42, 43, 47, 58, 68). This may be related to a perceived image of other PA participants being fitter, younger, slimmer or more beautiful (58, 68) and/or to the PA referral participants' own low self-esteem and body image (42, 47, 58). On scheme accessibility, participants expressed the following factors as barriers to adherence to the PARS programmes: Difficulty getting to programme sessions by public transport (29, 30, 58, 68), the time it takes to get to intervention venues (29), cost implications (59) and the perception of feeling unsafe (68). During the implementation of PARS, it is important that the administrators ensure that intervention venues are accessible and conducive for participants so as to optimize adherence to intervention and improved outcomes for participants.

Finally, timing and programme content were considered as major barriers. The timing for sessions was reported by some participants as unsuitable because they often coincided with work or childcare commitments, and as such, served as a barrier to attendance (29, 32, 46, 47, 49, 59, 68). Off-peak gym time programmes allowed attendance only when the environment was “less intimidating” but again, not suitable for day-time workers (47). Administrators of PARS should avoid “rigid” programme schedules as this could impact on uptake and attendance (47, 59).

In summary, the majority of the patients in the reviewed studies were referred for sedentary/inactivity related diseases and common group supervised activities was the most predominantly used intervention. Overall, the participants in the reviewed studies had a high adherence rate. This adherence was either facilitated or hindered by the type of support provided during and after the intervention period.

#### Strengths and Limitations

To the knowledge of the authors, this is the first multinational study on PARS, to examine six useful programme characteristics (disease conditions, intervention used, adherence/uptake, outcomes, facilitators and barriers) in order to explore the functionality of the PARS process holistically. Also, this review is the first to explore the outcome of PARS interventions, by categorizing diseases into similar groups. The findings of this review will aid healthcare providers in healthcare planning, enhancement of guidelines and advance insight into the most effective interventions for different chronic diseases. However, the findings of this review may have been limited by the search criteria. Predefined inclusion criteria were applied and although this ensured focus on the functionality of PARS, it resulted in the exclusion of several PA intervention studies. Also, the search criteria employed might have limited the total number of studies included in this review. The heterogeneity of the included studies and lack of methodological details in some of the studies could have potentially biased the review findings. Other limitations of this review are the selection of studies written in English language only and the fact that all the reviewed studies were from only developed countries. Nonetheless, the QATSDD assessment tool facilitated the assessment of studies with varying methodologies. This further strengthens the evidence from this review and showed that more medium based studies were assessed. The strengths of the analyzed studies depended on their aims/objectives, description of their research settings, how data was collected, the tools used, recruitment of participants and how the results were analyzed. Further improvements are required in describing theoretical frameworks, sample size, research question, and data collection methods.

### Conclusion

Findings from this review have highlighted that PARS process is, in itself, a key motivator and driver for individuals to take up and adhere to PA interventions. PARS should be considered for preventive medicine with early identification and referral of sedentary people to the PARS thereby curbing the proliferation of lifestyle diseases and their associated complications. Utilization of guidelines on evidence-based interventional PA for different types of diseases, effective use of common group supervised activities and the involvement of PA specialists may aid PA adherence and foster positive health outcomes. Finally, during the implementation of PARS process, administrators should be encouraged to focus on the professional and social on-going support available to participants, accessibility and conducive nature of the intervention venue/setting, as well as the timing and content of programme activities. Consideration of these factors could enhance the functionality of the PARS process and facilitate improved health outcomes for patients.

## Data Availability Statement

All datasets generated for this study are included in the article/Supplementary Material.

## Author Contributions

FA collected the data and developed the first draft of the manuscript. BM-A, MC, and AM-A advised on the data analysis and interpretation. All authors contributed to the article and approved the submitted version.

## Conflict of Interest

The authors declare that the research was conducted in the absence of any commercial or financial relationships that could be construed as a potential conflict of interest.

## References

[1] WHO Global Health Risks: Mortality and Burden of Disease Attributable to Selected Major Risks. Geneva: World Health Organization (2009).

[2] WHO Global Status Report on Noncommunicable Diseases 2014. Geneva: World Health Organization (2014).

[3] Biddle S Fox K Boutcher S Physical Activity and Psychological Well-Being. London; New York, NY: Routledge (2000/2003).

[4] Stathopoulou G Powers M Berry A Exercise interventions for mental health: a quantitativeand qualitative review. Clin Psychol Sci Pract. (2006) 13:179–93. 10.1111/j.1468-2850.2006.00021.x

[5] Williams HN Hendry M France B Lewis R Wilkinson C. Effectiveness of exercise-referral schemes to promote physical activity in adults: systematic review. Br J Gen Pract. (2007) 57:979–86. 10.3399/096016407782604866 18252074 PMC2084138

[6] Bully P Sanchez A Zabaleta-del-Olmo E Pombo H Grandes G. Evidence from interventions based on theoretical models for lifestyle modification (physical activity, diet, alcohol and tobacco use) in primary care settings: a systematic review. Prev Med. (2015) 76:S76–93. 10.1016/j.ypmed.2014.12.020 25572619

[7] National Institute for Health and Care Excellence Four Commonly Used Methods to Increase Physical Activity. London: Public Health Guidance [PH2] (2006).

[8] Orrow G Kinmonth A Sanderson S Sutton S. Effectiveness of physical activity promotion based in primary care: systematic review and meta-analysis of randomised controlled trials. BMJ. (2012) 344:16–9. 10.1136/bmj.e1389 22451477 PMC3312793

[9] US Preventive Services Task Force Behavioral counseling in primary care to promote physical activity: recommendations and rationale. Ann Intern Med. (2002) 137:205–7. 10.7326/0003-4819-137-3-200208060-00014 12160370

[10] Yarnall K Pollak K Østbye T Krause K Michener J. Primary care: is there enough time for prevention? Am J Public Health. (2003) 93:635–41. 10.2105/AJPH.93.4.635 12660210 PMC1447803

[11] Smith B van Der Ploeg H Buffart L Bauman A. Encouraging physical activity five steps for GPs. Aust Fam Physician. (2008) 37:24–8. 18239748

[12] Britt H Miller G Charles J Henderson J Bayram C Pan Y General Practice Activity in Australia BEACH: Bettering the Evaluation and Care of Health Sydney. Sydney, NSW: Sydney University Press (2016).

[13] Moyer VA Behavioral counseling interventions to promote a healthful diet and physical activity for cardiovascular disease prevention in adults: U.S. preventive services task force recommendation statement. Ann Intern Med. (2012) 157:367–72. 10.7326/0003-4819-157-5-201209040-00486 22733153

[14] Buchan J O'May F The Allied Health Professional Workforce: Evidence and Impact. East Lothian: Queen Margaret University Musselburgh (2011).

[15] James EL Ewald BD Johnson NA Stacey FG Brown WJ Holliday EG. Referral for expert physical activity counseling: a pragmatic RCT. Am J Prev Med. (2017) 53:490–9. 10.1016/j.amepre.2017.06.016 28818417

[16] Aittasalo M Miilunpalo S Kukkonen-Harjula K Pasanen M. A randomized intervention of physical activity promotion and patient self-monitoring in primary health care. Prev Med. (2006) 42:40–6. 10.1016/j.ypmed.2005.10.003 16297442

[17] Grandes G Sanchez A Sanchez-Pinilla RO Torcal J Montoya I Lizarraga K. Effectiveness of physical activity advice and prescription by physicians in routine primary care: a cluster randomized trial. Arch Intern Med. (2009) 69:694–701. 10.1001/archinternmed.2009.23 19364999

[18] Gademan MGJ Deutekom M Hosper K Stronks K. The effect of exercise on prescription on physical activity and wellbeing in a multi-ethnic female population: a controlled trial. BMC Public Health. (2012) 12:758. 10.1186/1471-2458-12-758 22963588 PMC3490781

[19] Karjalainen JJ Kiviniemi AM Hautala AJ Niva J Lepojärvi S Mäkikallio TH. Effects of exercise prescription on daily physical activity and maximal exercise capacity in coronary artery disease patients with and without type 2 diabetes. Clin Physiol Funct Imaging. (2012) 32:445–54. 10.1111/j.1475-097X.2012.01148.x 23031065

[20] Pavey TG Taylor AH Fox KR Hillsdon M Anokye N Campbell J. Effect of exercise referral schemes in primary care on physical activity and improving health outcomes: systematic review and meta-analysis. Br Med J. (2011) 343:1–14. 10.1136/bmj.d6462 22058134 PMC3209555

[21] Romé A Persson U Ekdahl C Gard G. Physical activity on prescription (PAP): costs and consequences of a randomized, controlled trial in primary healthcare. Scan J Prim Health Care. (2009) 27:216–22. 10.3109/02813430903438734 19929183 PMC3413913

[22] Morgan F Battersby A Weightman AL Searchfield L Turley R Morgan H. Adherence to exercise referral schemes by participants–what do providers and commissioners need to know? A systematic review of barriers and facilitators. BMC Public Health. (2016) 16:7. 10.1186/s12889-016-2882-7 26944952 PMC4779205

[23] Lundqvist S Börjesson M Larsson MEH Hagberg L Cider Å. Physical activity on prescription (PAP), in patients with metabolic risk factors. A 6-month follow-up study in primary health care. PLoS ONE. (2017) 12:e0175190. 10.1371/journal.pone.0175190 28403151 PMC5389642

[24] Sørensen J Skovgaard T Bredahl T Puggaard L. Exercise on prescription: changes in physical activity and health-related quality of life in five Danish programmes. Eur J Public Health. (2011) 21:56–62. 10.1093/eurpub/ckq003 20371500

[25] Vinson D Parke A Exercise service and support: client experiences of physical activity referral schemes (PARS). Qual Res Sport Exerc Health. (2012) 4:15–31. 10.1080/2159676X.2011.653501

[26] Foster MM Mitchell GK. ‘The onus is on me': primary care patient views of medicare? funded team care in chronic disease management in australia. Health Expect. (2015) 18:879–91. 10.1111/hex.12061 23521424 PMC5060831

[27] Lawlor D Hanratty B. The effect of physical activity advice given in routine primary care consultations: a systematic review. J Public Health Med. (2001) 23:219–26. 10.1093/pubmed/23.3.219 11585195

[28] Ackermann RT Deyo RA LoGerfo JP. Prompting primary providers to increase community exercise referrals for older adullts: a randomized trial. Am Geriatr Soc. (2005) 53:283–89. 10.1111/j.1532-5415.2005.53115.x 15673353

[29] James DVB Johnston LH Crone D Sidford AH Gidlow C Morris C. Factors associated with physical activity referral uptake and participation. J Sports Sci. (2008) 26:217–24. 10.1080/02640410701468863 17943595

[30] Moore GF Raisanen L Moore L Din NU Murphy S Mixed-method process evaluation of the welsh national exercise referral scheme. Health Educ. (2013) 113:476–501. 10.1108/HE-08-2012-0046

[31] Cant R Foster M. Investing in big ideas: utilisation and cost of medicare allied health services in australia under the chronic disease management initiative in primary care. Austr Health Rev. (2011) 35:468–74. 10.1071/AH10938 22126951

[32] Dugdill L Graham RC McNair F. Exercise referral: the public health panacea for physical activity promotion? A critical perspective of exercise referral schemes; their development and evaluation. Ergonomics. (2005) 48:1390–410. 10.1080/00140130500101544 16338708

[33] Shore CB Hubbard G Gorely T Polson R Hunter A Galloway SD. Insufficient reporting of factors associated with exercise referral scheme uptake, attendance, and adherence: a systematic review of reviews. J Phys Act Health. (2019) 16:667–76. 10.1123/jpah.2018-0341 31203705

[34] Duda J Williams G Ntoumanis N Daley A Eves F Mutrie N. Effects of a standard provision versus an autonomy supportive exercise referral programme on physical activity, quality of life and well-being indicators : a cluster randomised controlled trial. Int J Behav Nutr Phys Act. (2014) 11:10. 10.1186/1479-5868-11-10 24475766 PMC3931311

[35] Edwards RT Linck P Hounsome N Raisanen L Williams N Moore L. Cost-effectiveness of a national exercise referral programme for primary care patients in wales: results of a randomised controlled trial. BMC Public Health. (2013) 13:1021. 10.1186/1471-2458-13-1021 24164697 PMC4231449

[36] Romé A Persson U Ekdahl C Gard G Costs and outcomes of an exercise referral programme-A 1-year follow-up study. Eur J Physiother. (2014) 16:82–92. 10.3109/21679169.2014.886291

[37] Moher D Liberati A Tetzlaff J Altman D The PRISMA Group. Preferred reporting items for systematic reviews and meta-analyses: the PRISMA statement. J Clin Epidemiol. (2009) 62:1006–12. 10.1016/j.jclinepi.2009.06.005 19631508

[38] Moher D Liberati A Tetzlaff J Altman DG PRISMA Group Preferred reporting items for systematic reviews and meta-analyses: the PRISMA statement. Intern J Surg. (2010) 8:336–41. 10.1016/j.ijsu.2010.02.007 20171303

[39] Vaismoradi M Turunen H Terese B. Content analysis and thematic analysis: implications for conducting a qualitative descriptive study. Nurs Health Sci. (2013) 15:398–405. 10.1111/nhs.12048 23480423

[40] Sirriyeh R Lawton R Gardner P Armitage G. Reviewing studies with diverse designs: the development and evaluation of a new tool. J Eval Clin Pract. (2012) 18:746–52. 10.1111/j.1365-2753.2011.01662.x 21410846

[41] Isaacs AJ Critchley JA Tai SS Buckingham K Westley D Harridge SD. Exercise evaluation randomised trial (EXERT): a randomised trial comparing GP referral for leisure centre-based exercise, community-based walking and advice only. Health Technol Assess. (2007) 11:1–165. 10.3310/hta11100 17313906

[42] Joyce K Smith KE Henderson G Greig G Bambra C. Patient perspectives of condition management programmes as a route to better health, wellbeing and employability. Family Pract. (2010) 27:101–9. 10.1093/fampra/cmp083 19948563

[43] Wormald H Waters H Sleap M Ingle L. Participants' perceptions of a lifestyle approach to promoting physical activity: targeting deprived communities in Kingston-Upon-Hull. BMC Public Health. (2006) 6:202. 10.1186/1471-2458-6-202 16889657 PMC1560127

[44] Eynon MJ O'Donnell C Williams L. Gaining qualitative insight into the subjective experiences of adherers to an exercise referral scheme: a thematic analysis. J Health Psychol. (2018) 23:1476–87. 10.1177/1359105316656233 27387512

[45] Hanson CL Oliver EJ Dodd-Reynolds CJ Allin LJ. How do participant experiences and characteristics influence engagement in exercise referral? A qualitative longitudinal study of a scheme in Northumberland, UK. BMJ Open. (2019) 9:e024370. 10.1136/bmjopen-2018-024370 30787087 PMC6398729

[46] Sørensen JP Kragstrup J Skovgaard T Puggaard L. Exercise on prescription: a randomized study on the effect of counseling vs counseling and supervised exercise. Scand J Med Sci Sports. (2008) 18:288–97. 10.1111/j.1600-0838.2008.00811.x 18503642

[47] Taylor A Doust J Webborn N. Randomised controlled trial to examine the effects of a GP exercise referral programme in Hailsham, East Sussex, on modifiable coronary heart disease risk factors. J Epidemiol Community Health. (1998) 52:595–601. 10.1136/jech.52.9.595 10320861 PMC1756762

[48] Law R-J Nafees S Hiscock J Wynne C Williams NH. A lifestyle management programme focused on exercise, diet and physiotherapy support for patients with hip or knee osteoarthritis and a body mass index over 35: a qualitative study. Musculoskeletal Care. (2019) 17:145–51. 10.1002/msc.1382 30677219

[49] Dinan S Lenihan P Tenn T Iliffe S. Is the promotion of physical activity in vulnerable older people feasible and effective in general practice? Br J Gen Pract. (2006) 56:791–3. 17007711 PMC1920721

[50] Sharma H Bulley C van Wijck FMJ. Experiences of an exercise referral scheme from the perspective of people with chronic stroke: a qualitative study. Physiotherapy (United Kingdom). (2012) 98:341–8. 10.1016/j.physio.2011.05.004 23122441

[51] Grandes G Sanchez A Montoya I Sanchez-Pinilla RO Torcal J. Two-year longitudinal analysis of a cluster randomized trial of physical activity promotion by general practitioners. PLoS ONE. (2011) 6:e18363. 10.1371/journal.pone.0018363 21479243 PMC3066231

[52] Martín-Borràs C Giné-Garriga M Puig-Ribera A Martín C Solà M Cuesta-Vargas AI A new model of exercise referral scheme in primary care: is the effect on adherence to physical activity sustainable in the long term? A 15-month randomised controlled trial. BMJ Open. (2018) 8:e017211 10.1136/bmjopen-2017-017211 PMC5855315 29502081

[53] Gusi N Reyes MC Gonzalez-Guerrero JL Emilio H Jose MG Cost-utility of a walking programme for moderately depressed, obese, or overweight elderly women in primary care: a randomised controlled trial. BMC Fam Pract. (2008) 8:231 10.1186/1471-2458-8-231 PMC2491610 18611277

[54] Livingston PM Craike MJ Salmon J Courneya KS Gaskin CJ Fraser SF. Effects of a clinician referral and exercise program for men who have completed active treatment for prostate cancer: a multicenter cluster randomized controlled trial (ENGAGE). Cancer. (2015) 121:2646–54. 10.1002/cncr.29385 25877784 PMC4654333

[55] Kallings LV Leijon ME Kowalski J Hellénius M Ståhle A. Self-reported adherence: a method for evaluating prescribed physical activity in primary health care patients. J Phys Act Health. (2009) 6:483–92. 10.1123/jpah.6.4.483 19842463

[56] Roessler KK Ibsen B Promoting exercise on prescription: recruitment, motivation, barriers and adherence in a danish community intervention study to reduce type 2 diabetes, dyslipidemia and hypertension. J Public Health. (2009) 17:187–93. 10.1007/s10389-008-0235-4

[57] Patel A Schofield GM Kolt GS Keogh JW. Perceived barriers, benefits, and motives for physical activity: two primary-care physical activity prescription programs. J Aging Phys Act. (2013) 21:85–99. 10.1123/japa.21.1.85 22832475

[58] Wormald H Ingle L GP exercise referral schemes: improving the patient's experience. Health Educ J. (2004) 63:362–73. 10.1177/001789690406300407

[59] Shaw R Gillies M Barber J MacIntyre K Harkins C Findlay IN. Pre-exercise screening and health coaching in CHD secondary prevention: a qualitative study of the patient experience. Health Educ Res. (2012) 27:424–36. 10.1093/her/cys005 22313621

[60] Rowley N Mann S Steele J Horton E Jimenez A. The effects of exercise referral schemes in the United Kingdom in those with cardiovascular, mental health, and musculoskeletal disorders: a preliminary systematic review. BMC Public Health. (2018) 18:949. 10.1186/s12889-018-5868-9 30068338 PMC6090762

[61] Wisdom JP Cavaleri MA Onwuegbuzie AJ Green CA. Methodological reporting in qualitative, quantitative, and mixed methods health services research articles. Health Serv Res. (2012) 47:721–45. 10.1111/j.1475-6773.2011.01344.x 22092040 PMC3419885

[62] Creswell JW Plano Clark VL Designing and Conducting Mixed Methods Research 3rd ed Thousand Oaks, CA: Sage Publications (2017).

[63] Leijon ME Bendtsen P Nilsen P Ekberg K Ståhle A. Physical activity referrals in Swedish primary health care–prescriber and patient characteristics, reasons for prescriptions, and prescribed activities. BMC Health Serv Res. (2008) 8:201. 10.1186/1472-6963-8-201 18828898 PMC2567971

[64] Pavey T Anokye N Taylor A Trueman P Moxham T Fox K The clinical effectiveness and cost-effectiveness of exercise referral schemes: a systematic review and economic evaluation. Health Technol Assess. (2011) 15:1–254. 10.3310/hta15440 PMC4781450 22182828

[65] Hanson C Neubeck L Dodd-Reynolds CJ A physical activity referral program improves risk factors in those who have completed cardiac rehabilitation. Heart Lung Circ. (2017) 26:S342–3. 10.1016/j.hlc.2017.06.695

[66] Saunders DH Greig CA Mead GE. Physical activity and exercise after stroke: Review of multiple meaningful benefit. Stroke. (2014) 45:3742–7. 10.1161/STROKEAHA.114.004311 25370588

[67] Spence RR Heesch KC Brown WJ. Exercise and cancer rehabilitation: a systematic review. Cancer Treat Rev. (2010) 36:185–94. 10.1016/j.ctrv.2009.11.003 19962830

[68] Martin C Woolf-May K The retrospective evaluation of a general practitioner exercise prescription programme. J Hum Nutr Diet. (1999) 12:32–42. 10.1046/j.1365-277X.1999.00005.x

[69] Beers H Factors Influencing Physical Activity Behaviour in Adults at Risk of Coronary Heart Disease: A Quantitative and Qualitative Study of an Exercise Referral Scheme. Liverpool: University of Liverpool (2006).

[70] Wiles R Demain S Robison J Kileff J Ellis-Hill C McPherson K. Exercise on prescription schemes for stroke patients post-discharge from physiotherapy. Disabil Rehabil. (2008) 30:1966–75. 10.1080/09638280701772997 18608413

