# Supplementary material for: Functionality of Physical Activity Referral Schemes (PARS): A Systematic Review
Source: Front Public Health. 2020 Jun 25;8:257. doi: 10.3389/fpubh.2020.00257 (PMC7329989; doi:10.3389/fpubh.2020.00257)
Supplement: Supplementary file 1 [file Data_Sheet_1.docx]

Appendix 1. STUDY SEARCH TERMS

exercise* OR "physical activit*" OR sport* OR walk* OR run* OR "physical fitness" OR exertion OR "exercise on referral" OR "physical activity on prescription" OR "exercise on prescription" OR "medicine is exercise" OR "green prescription" OR “exercise referral scheme” OR “physical activity promotion”

AND
“general practice physician” OR “general practi*" OR "family physician*" OR "family practi*" OR "family doctor*" OR gp OR "home doctor*" OR generalists

AND
"physical therap*" OR physio* OR "exercise physiolog*" OR "physical trainer*" OR "personal trainer*" OR "fitness train*" OR "fitness instruct*” OR “health personnel” OR “primary care” OR “primary healthcare” OR “patient care team” OR “Integrated healthcare” OR “integrated health care” OR “patient care team*” OR “allied health p*”

AND
refer* OR "secondary car*" OR transfer OR send OR "consultation, referral" OR "health service gatekeeper*" OR "second opinion" OR consult*

AND
"life style*" OR "life style induced illness*" OR sedentary OR “sedentary behaviour” OR "health behavio*" OR "lifestyle disease*" OR "life style, sedentary" OR "life style change*" OR barrier* OR facilitat*
