# Supplementary material for: Functionality of Physical Activity Referral Schemes (PARS): A Systematic Review
Source: Front Public Health. 2020 Jun 25;8:257. doi: 10.3389/fpubh.2020.00257 (PMC7329989; doi:10.3389/fpubh.2020.00257)
Supplement: Supplementary file 2 [file Data_Sheet_2.docx]

**Appendix 2: Summary of study characteristics**

| **Author & Year** | **Aim of study** | **Country of study** | **Study design** | **Setting (Venue & PA specialist)** | **Participants (No., Gender, Mean Age (yrs.)** |
| --- | --- | --- | --- | --- | --- |
| Ackermann et al^28^ (2005) | Using clinic-based PA exercise to increase motivations. | USA | RCT | Clinic  Intervention conducted by physicians, nurse practitioners and physician assistants | N = 336, Male (99%, n = 332),  Age: (66.0+4) |
| Dinan et al^57^ (2006) | Feasibility and effectiveness of referral programs for frail elderly patients | UK | Prospective cohort studies | Varied (Clinic, Leisure centre & primary health care)  Specialist exercise instructors led intervention | N = 242 Gender*  Age: (82.0+4.6) |
| Dugdill et al^32^ (2005) | Leisure time PA levels | UK | 2 Case evaluation studies | Leisure centre  Exercise referral officer (ERO) led intervention | N = 958 Males (n = 344)  Age: (44.0+15.7) |
| Eynon et al^50^ (2018) | To uncover the key psychological factors associated with adherence to the scheme based on adherers’ subjective experiences, with a particular focus on motivational experiences while also taking into account any other pertinent factors driven by the participants | UK | Qualitative | Leisure centre  Intervention led by exercise specialists | N = 9 Males (44.4%, n = 4)  Age: (49.9+8.6) |
| Gademan et al^18^ (2012) | To evaluate the effect of EoP in physical inactive women living in multi-ethnic neighbourhoods in the Netherlands | Netherlands | RCT | Leisure centre  Sports instructor led intervention | N =514 All females  Age: (45.0+10) |
| Grandes et al^42^ (2011) | Evaluate the effectiveness of a PA promotion programme | Spain | RCT | Primary health care  GP led intervention | N = 3,691 Male (35.4%, n=1,307) Age: (50.3+14.66) |
| Grandes et al^17^ (2009) | Assess the effectiveness of a PA on prescription | Spain | RCT | Primary health care  Physician led intervention | N = 4,317 Male (34.3%, n = 1,484) Age: (49.5+14.88) |
| Gusi et al^51^ (2008) | Assesses the cost utility of the adding a supervised walking programme to the standard | Spain | RCT | Varied (Clinic, Leisure centre & primary health care)  Qualified exercise leader led intervention | N = 127 All female  Age: (72.6+1.5) |
| Hanson et al^52^ (2019) | To gain an insight into differential engagement through understanding participants experiences of patients referred for ERS | UK | Qualitative | Leisure centres | N = 15 Males (27%, n = 4)  Age* |
| Isaacs et al^43^ (2007) | To evaluate and compare the effectiveness and cost-effectiveness of a leisure centre-based exercise programme, walking program and advice-only | UK | RCT | Leisure centre  Fitness instructor led intervention | N = 943 Males (32.7% n =308)  Age: (57.0+8.73) |
| James et al^15^ (2017) | Determine the efficacy of primary care physicians’ referral | Australia | RCT | Primary health care  Accredited exercise physiologist (AEPs) led intervention | N = 203 Males (29.5% n =60)  Age: (57.0+13.0) |
| Joyce et al^44^ (2010) | Explore patients’ experiences of condition management programmes | UK | Qualitative | Primary health care  Intervention led by intervention leaders | N = 25; Males (40%, n = 10);  Age: (= >50 years) |
| Kallings et al^45^ (2009) | Examine self-reported adherence to individualized prescribed PA | Sweden | Prospective study | Primary care centre  Self-reported physical activity by participants | N = 240 Males (25% n = 60)  Age: (51.0+13.0) |
| Law et al^56^ (2019) | To explore views and experiences of lifestyle management programme (LMP) among patients and professionals | UK | Qualitative | Home-based/telephone interviews  Physiotherapist, exercise professionals and dietitians | N = 9 Males (22% n = 2)  Age: (58.0+13.0) |
| Livingstone et al^59^ (2015) | To determine the efficacy of a clinician referral and exercise programmed in improving exercise levels and quality of life for men with prostate cancer | Australia | RCT | Local community gym  Exercise physiologist | N = 147 All males  Age: (65.6+8.5) |
| Lundqvist et al^23^ (2017) | Explore the association between PA on prescription treatment and the PA level of patients with metabolic risk | Sweden | Prospective/longitudinal observational study. | Health care centres  A nurse led intervention | N = 368 Males (42.6% n = 170)  Age: (57.4+13.0) |
| Martin-Borras et al^46^ (2018) | To assess the effectiveness and sustainability of a primary care-based ERS | Spain | RCT | Leisure centre  PA specialist led intervention | N = 422; Males (39.1%, n = 165)  Age: (68.8+8.65) |
| Moore et al^30^ (2013) | Mixed-method process evaluation exploring how outcomes were achieved | UK | Mixed methods | Leisure centre  Exercise specialist led intervention | N = 1080 Males (34.5%, n = 373) Age: (50. 3) |
| Patel et al^49^ (2013) | To examine whether perceived barriers, benefits, and motives for physical activity differed based on allocation to 2 different types of primary-care activity- prescription programs (pedometer-based vs. time-based Green Prescription) | New Zealand | Quantitative (Cross sectional studies) | Primary Health Care  Physician led intervention | N = 80 Males (40%, n = 32)  Age:* |
| Roessler & Ibsen^47^ (2009) | Analyse recruitment, motivation, barriers and adherence to increasing PA | Denmark | Longitudinal observational study | Primary health care  Physiotherapist and dietician led intervention | N = 1156 Male (33%, n = 382),  Age: (50.5+12.3) |
| Romé et al^36^ (2014) | Analyse 1-year follow up, of cost offset and outcomes of changing the PA behaviour | Sweden | RCT | Primary health care  Physiotherapist led intervention | N = 528 Males 23% (n = 123).  Age: (53.2+12.7) |
| Sharma et al^70^ (2012) | To explore stroke survivors experience of undertaking ERS | UK | Qualitative | Varied  Physiotherapist led intervention | N = 9 Males (55.5%, n = 5)  Age: (51.0+ 9.0) |
| Shaw et al^60^ (2012) | To explore patients’ attitudes towards, and experiences of a lifestyle intervention | UK | Qualitative | Varied  Health coach led intervention | N = 84 Males (48.8%, n = 41.  Age: (66.9+10.4) |
| Sorensen et al^54^ (2008) | To compare short and long-term effects of high-intensive exercise on prescription and low intensive intervention in primary care | Denmark | RCT | Primary health care  Physiotherapist led intervention | N = 52 Gender* Age: (53.4) |
| Taylor et al^55^ (1998) | Examine the effects of a GP exercise referral programme | UK | RCT | Health and leisure centre  Trained assessor led intervention | N = 142 Males (57%, n = 82)  Age: (54.5+ 0.46) |
| Wormald & Ingle^58^ (2004) | To explore participant’s experiences of ERS | UK | Qualitative | Leisure centre  Leisure centre staff led intervention | N = 30 Males (33.3%, n = 10)  Age: (54.5+18.0) |
| Wormald et al^48^ (2006) | Explore participants' perceptions of the operation and effectiveness of the active lifestyle (AL) service | UK | Qualitative | Leisure centre  Intervention led by active lifestyle (AL) advisor | N = 16 Male (31.25%, n = 5)  Age: (53+12) |

*Not indicated
